# Cardiovascular risk assessment in inflammatory bowel disease with coronary calcium score

**DOI:** 10.1097/MS9.0000000000001652

**Published:** 2024-01-04

**Authors:** Waqar Arif Rasool Chaudhry, Muhammad Ashfaq, Parvinder Kaur, Mahendra Kumar, Maria Faraz, Jahanzeb Malik, Amin Mehmoodi

**Affiliations:** a Department of Cardiovascular Medicine, Cardiovascular Analytics Group, Islamabad, Pakistan; b Department of Medicine, Ibn e Seena Hospital, Kabul, Afghanistan

**Keywords:** atherosclerotic cardiovascular disease, chronic inflammation, coronary calcium score, inflammatory bowel disease, risk assessment

## Abstract

The interplay between inflammatory bowel disease (IBD) and atherosclerotic cardiovascular disease (ASCVD) underscores the intricate connections between chronic inflammation and cardiovascular health. This review explores the multifaceted relationship between these conditions, highlighting the emerging significance of the coronary calcium score as a pivotal tool in risk assessment and management. Chronic inflammation, a hallmark of IBD, has far-reaching systemic effects that extend to the cardiovascular system. Shared risk factors and mechanisms, such as endothelial dysfunction, lipid dysfunction, and microbiome dysregulation, contribute to the elevated ASCVD risk observed in individuals with IBD. Amidst this landscape, the coronary calcium score emerges as a means to quantify calcified plaque within coronary arteries, offering insights into atherosclerotic burden and potential risk stratification. The integration of the coronary calcium score refines cardiovascular risk assessment, enabling tailored preventive strategies for individuals with IBD. By identifying those at elevated risk, healthcare providers can guide interventions, fostering informed shared decision-making. Research gaps persist, prompting further investigation into mechanisms linking IBD and ASCVD, particularly in the context of intermediate mechanisms and early atherosclerotic changes. The potential of the coronary calcium score extends beyond risk assessment—it holds promise for targeted interventions. Randomized trials exploring the impact of IBD-modifying therapies on ASCVD risk reduction can revolutionize preventive strategies. As precision medicine gains prominence, the coronary calcium score becomes a beacon of insight, illuminating the path toward personalized cardiovascular care for individuals living with IBD. Through interdisciplinary collaboration and rigorous research, we embark on a journey to transform the paradigm of preventive medicine and enhance the well-being of this patient population.

## Introduction

HIGHLIGHTS

- The interplay between inflammatory bowel disease (IBD) and atherosclerotic cardiovascular disease underscores the intricate connections between chronic inflammation and cardiovascular health.
- Chronic inflammation is a hallmark of IBD.
- It has far-reaching systemic effects that extend to the cardiovascular system.
- Shared risk factors and mechanisms, such as endothelial dysfunction, lipid dysfunction, and microbiome dysregulation, contribute to the elevated ASCVD risk observed in individuals with IBD.
- The integration of the coronary calcium score refines cardiovascular risk assessment, enabling tailored preventive strategies for individuals with IBD.

Atherosclerotic cardiovascular disease (ASCVD) remains the leading global cause of death, emphasizing the need for intensified preventive measures, especially among young adults^1^. Certain characteristics prevalent in young and middle-aged individuals, like chronic inflammatory diseases, are autonomously linked with the onset of ASCVD^2,3^. These attributes have been gaining attention as potential focal points for personalized and timely preventive interventions. Chronic inflammation significantly increases the risk of atherosclerosis, thrombosis, and ASCVD events^4–6^. Individuals with conditions like human immunodeficiency virus (HIV) infection, psoriasis, and rheumatoid arthritis experience expedited atherosclerosis^3–5^. These patients exhibit higher levels of concealed carotid and coronary plaque, more frequent high-risk plaque traits, and heightened ASCVD event rates compared to healthy counterparts^7–10^. Recent studies such as CANTOS (Canakinumab Anti-Inflammatory Thrombosis Outcome Study) and COLCOT (Colchicine Cardiovascular Outcomes Trial) have provided compelling evidence supporting the causal relationship between systemic inflammation and ASCVD^11,12^.

Inflammatory bowel disease (IBD) constitutes a chronic condition encompassing ulcerative colitis (UC), Crohn’s disease (CD), and indeterminate colitis^13^. These conditions entail persistent inflammation in the digestive tract, often accompanied by elevated systemic inflammation and other non-digestive symptoms. Globally, IBD affects ~6.8 million individuals^14^. Comprehensive analyses of multiple studies have unveiled connections between IBD and ASCVD, even after accounting for conventional risk factors^15–17^. This intricate interplay is further fuelled by disruptions in the gut microbiome and the frequent use of steroids in IBD patients.

Preventive cardiologists face notable challenges in managing cardiovascular risk among patients with IBD. These challenges include the scarcity of specific guidance and the inadequacy of widely used clinical risk assessment tools to accurately gauge their elevated ASCVD risk. To elucidate these aspects, this review expounds upon the epidemiological associations linking IBD with ASCVD and the potential underlying mechanisms. Moreover, it delves into the role of chronic inflammatory conditions within existing primary prevention guidelines and targeted strategies tailored to reduce ASCVD risk specifically in IBD patients. Finally, the review outlines current gaps in knowledge and lays out prospective directions for further exploration in this field.

## Methodology

A comprehensive search strategy was devised to identify relevant studies and articles from electronic databases such as PubMed, MEDLINE, Scopus, and Google Scholar. A combination of keywords and Medical Subject Headings (MeSH) terms, including “Inflammatory Bowel Disease,” “Cardiovascular Risk,” “Coronary Calcium Score,” and related synonyms, was used to ensure the inclusiveness of the search. The initial search yielded a considerable number of potentially relevant articles. Following the search, a two-stage screening process was implemented to select the most relevant studies. In the first stage, titles and abstracts were screened to exclude articles that were clearly unrelated to the topic. In the second stage, the full texts of the remaining articles were assessed for eligibility based on predefined inclusion and exclusion criteria. The synthesis of findings involved a narrative approach, aiming to provide a cohesive and holistic view of the current state of knowledge on the topic. In the absence of meta-analysis, a qualitative analysis of the evidence was carried out, discussing the strengths and limitations of individual studies, as well as identifying common trends and discrepancies in the results.

## Basics of IBD

### Epidemiology

On a global scale, the impact of IBD is substantial, affecting more than 6.8 million individuals^14^. This prevalence has seen an upward trajectory due to decreased mortality rates, with the incidence remaining stable or even declining in regions like North America and Europe^18^. However, an interesting shift has been observed since 1990, with an increasing incidence of IBD in large, newly industrialized nations across Africa, Asia, and South America^18^. In the year 2017, the worldwide age-standardized prevalence of IBD was noted at 84.3 cases per 100 000 population, marking an increase from 79.5 cases per 100 000 in 1990^14^.

Among specific countries, the United States exhibited the highest age-standardized prevalence at 464.5 cases per 100 000, followed closely by the United Kingdom at 449.6 cases per 100 000^14^. Of particular significance is the observation that the peak onset of IBD generally occurs between the second and fourth decades of an individual’s life. This means that those affected by IBD experience prolonged and continuous exposure to chronic systemic inflammation throughout a significant portion of their lives. This chronic inflammation, inherent to IBD, can have extensive ramifications on various aspects of health, potentially leading to an array of complications and comorbidities^14^.

The evolving global landscape of IBD highlights its increasing significance as a public health concern, with its prevalence shifting across regions and the burden of the disease extending over extended periods in affected individuals’ lives^15^. Understanding these dynamics is pivotal for the development of effective management strategies and interventions to alleviate the burden of chronic inflammation and its associated consequences in IBD patients.

### Etiology

IBD is believed to stem from intricate interactions among genetic susceptibility, the environment, microbiota, and the immune system, all converging in genetically predisposed individuals. Researchers have identified over 200 genetic regions associated with the risk of developing IBD, many of which are linked to immune system functioning^19^. One of these genes, nucleotide oligomerization domain 2 (NOD2), encodes an intracellular pattern recognition receptor that plays a crucial role in safeguarding against intracellular bacteria and orchestrating immune responses to microbes that inhabit the body in a mutually beneficial manner^20,21^.

When NOD2 mutations occur, they lead to reduced levels of an anti-inflammatory cytokine called interleukin (IL)–10 and an increase in the number of bacteria found in the mucosa of the intestines^22^. Multiple environmental factors have been implicated in the development of IBD. Certain aspects of urban living, such as residing in urban areas, along with medical procedures like appendectomy and tonsillectomy, consumption of sugary soft drinks, and the use of antibiotics have been identified as potential risk factors^23^.

Interestingly, smoking has been established as a risk factor for CD, one of the subtypes of IBD. However, it seems to confer a protective effect against UC, another subtype of IBD, although the precise mechanisms behind this phenomenon are not yet fully elucidated^24,25^. On the other hand, certain factors appear to be associated with a decreased risk of developing IBD. These include engaging in regular physical activity, being breastfed during infancy, and having an infection with Helicobacter pylori, a type of bacteria found in the stomach associated with reduced risk for IBD^23^. Thus far, one of the most robust risk factors identified for IBD is having a positive family history of the disease. Individuals with close relatives who have IBD are at a significantly higher risk of developing the condition themselves^26^. This underscores the influence of genetic predisposition in conjunction with the interplay of environmental and lifestyle factors in the complex etiology of IBD. Gaining a deeper understanding of these multifaceted interactions is crucial for not only elucidating the mechanisms underlying IBD but also for devising effective strategies for the prevention, treatment, and management of the disease.

### Mechanism

The microbiome, the collection of microorganisms inhabiting the body, undergoes significant alterations in individuals with IBD when compared to healthy individuals^27^. In fact, specific microbial patterns have been identified that can differentiate between stool samples of patients with CD and those without the condition^28^. Generally, there’s a reduction in species belonging to the Firmicutes group and an increase in species from Proteobacteria. Additionally, the gut microbiota in individuals with IBD is characterized by lower diversity and greater instability over time in contrast to the more stable and diverse microbiota in healthy subjects^29^. However, there’s ongoing debate regarding whether these microbial changes observed in IBD are a cause or a consequence of inflammation.

**Figure 1:**
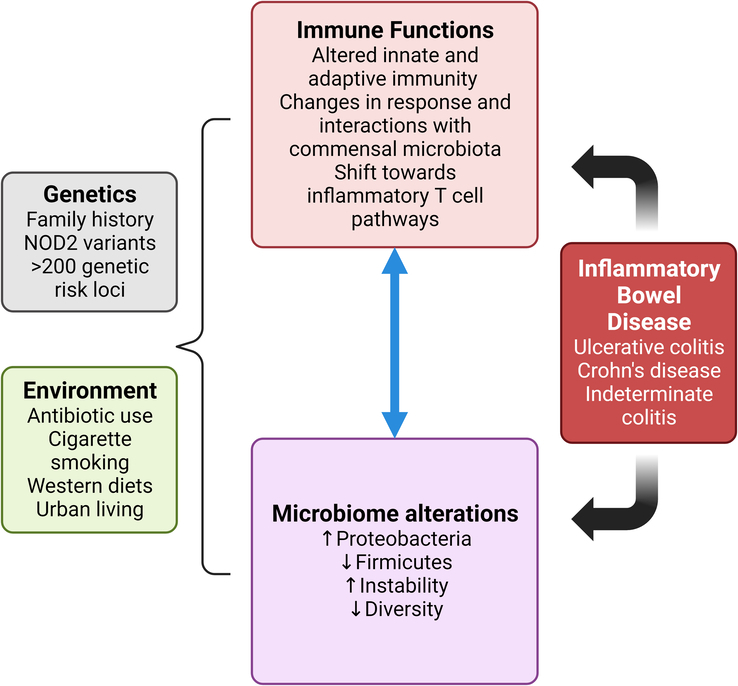
The mechanism involved in the genesis of inflammatory bowel disease.

Notable modifications occur in both the innate and adaptive immune systems in IBD^30^. In terms of innate immunity, changes encompass alterations in signalling among antigen-presenting cells, regulation of intracellular bacteria, and the production of substances like defensins, leading to an exaggerated response by the adaptive immune system to various types of antigens, including those from the diet, commensal organisms (microbes that normally reside in the body), and self-antigens. This ultimately culminates in inflammation. Within IBD, macrophages in the mucosal lining secrete increased levels of proinflammatory cytokines such as IL-1β, IL-6, IL-23, and tumour necrosis factor (TNF), which subsequently stimulate local mononuclear cells to produce interferon-γ^31^. Dendritic cells, which play a pivotal role in initiating immune responses, exhibit heightened levels of Toll-like receptors 2 and 4 and generate more proinflammatory cytokines like IL-12 and IL-6^32^. Changes in the IL-12/IL-23 pathway lead to a shift in the immune response towards inflammatory pathways involving specific types of T-helper (Th) cells, particularly Th17 and Th1 responses^33^. Th17 cells produce IL-17, a potent proinflammatory cytokine, which when specifically targeted in conditions like psoriasis, has been shown to reduce coronary plaque^34,35^. The Th1 response involves the production of cytokines like TNF and interferon-γ, which are known to have synergistic effects in promoting the development of atherosclerosis and cholesterol crystals, particularly notable in conditions like psoriasis^36,37^. The release of cytokines from these proinflammatory T-cell subsets is thought to be intricately connected to disruptions in the integrity of the epithelial barrier lining the intestines, heightened expression of endothelial adhesion molecules, and increased collagen production. This cascade of events ultimately leads to inflammation and tissue damage^35^. Figure 1 shows the key mechanism involved in the genesis of IBD.

### Clinical course

In addition to symptoms primarily affecting the gastrointestinal tract, IBD has a systemic nature, with around 40% of patients experiencing manifestations beyond the intestines. It’s noteworthy that individuals with IBD commonly go through periods of active symptomatic flares, characterized by heightened inflammation, followed by extended periods of remission, during which the inflammatory activity is considerably low.

### The link between IBD and CVD

Multiple comprehensive analyses, including two recent meta-analyses pooling data from up to 27 cohort studies, substantiate the existence of an independent link between IBD and subsequent ASCVD^15,16^. To illustrate, a meta-analysis conducted in 2017, encompassing data from 10 cohort studies, demonstrated a statistically adjusted and independent connection between IBD and the occurrence of CHD, with a combined relative risk (RR) of 1.24 and a 95% CI of 1.14–1.36. This relationship remained consistent when focusing on subgroups of patients with either Crohn’s disease (CD) or ulcerative colitis (UC)^15^. A subsequent meta-analysis, involving a larger dataset, reinforced these findings. It highlighted a consistent association between IBD and not only CHD (pooled RR: 1.17; 95% CI: 1.07–1.27) but also myocardial infarction (RR: 1.12; 95% CI: 1.05–1.21) and the onset of cerebrovascular disease (RR: 1.25; 95% CI: 1.08–1.44). Furthermore, these associations appeared to be more pronounced among women^16^. These meta-analyses serve to underscore conclusions drawn from significant cohort studies, such as a nationwide study conducted in Denmark encompassing 4.3 million individuals, including 28 833 with IBD^17^. This research, which followed participants for up to 13 years, unveiled a correlation between IBD and CHD events, even after accounting for multiple potential confounding factors.

A more recent U.S. study, utilizing cross-sectional data from 26 nationwide healthcare systems and involving roughly 290 000 individuals aged 20 to 65 with IBD, reinforced these findings. It observed a robust connection between IBD and a history of myocardial infarction, with an adjusted odds ratio of 1.25 (95% CI: 1.24–1.27)^38^. This relationship appeared to be stronger among younger age groups. It is important to acknowledge, however, that findings from cross-sectional studies exhibit variability. Yet, many of these studies were accompanied by limitations, including their inability to establish temporal relationships and often the restriction of study populations to hospitalized patients^39^. Research also proposes that heightened cardiovascular risk in IBD is particularly associated with active disease states^40^. A Danish study involving 20 795 patients demonstrated a significantly elevated risk for myocardial infarction, stroke, and cardiovascular death during IBD flares, whereas during remission, the risk was comparable to that of control subjects^40^. Additionally, the risk for ASCVD seems to be especially elevated within the first year following IBD diagnosis, likely due to disease activity. Moreover, IBD is linked to a 2-fold to 3-fold higher risk for venous thromboembolic events, especially during acute flares, presumably stemming from a state of increased blood clotting propensity^41–43^.

Increased risk for mesenteric ischaemia, a condition involving reduced blood supply to the intestines, has also been identified in meta-analyses^44^. However, contrary to these associations, there has been no observed connection between IBD and cardiovascular mortality^16,43,45^. This lack of association might potentially be explained by factors, such as the relatively young age of the IBD population and the relatively low case-fatality rates of ASCVD events within this age group. Nonetheless, more extensive research is necessary to fully grasp this apparent lack of connection.

### Mechanism of IBD-CVD link

Numerous ongoing processes that are persistently active in individuals with IBD have been implicated in the development of ASCVD. These processes encompass both local and systemic levels of inflammation, abnormalities in the gut microbiome, compromised endothelial function, thrombosis, disturbances in lipid metabolism, and the detrimental effects associated with certain IBD treatments, notably corticosteroids^17,27,46–50^. Corticosteroids have been linked to insulin resistance, hypertension, and dyslipidemia. However, further research is imperative in this realm, focusing on understanding the relative significance of each mechanism, discerning potential divergences from mechanisms associated with other chronic inflammatory conditions, and identifying novel mechanisms that could serve as targets for interventions aimed at reducing ASCVD risk^13^.

Interestingly, IBD and ASCVD also share some underlying risk factors, which expose patients to vulnerability for both conditions^13^. For instance, modern Western lifestyles have been associated with the onset of both IBD and ASCVD. Chronic stress has been linked to elevated levels of inflammatory markers such as high-sensitivity C-reactive protein and IL-6^51^. In other inflammatory diseases like psoriasis, heightened neural activity due to stress correlates with vascular inflammation and the development of coronary plaque. Anxiety and mental stress play roles in the development of ASCVD, and in individuals genetically predisposed to IBD, they are also linked with the onset of the condition^52,53^. Moreover, chronic stress has been linked to the occurrence of symptomatic flares in IBD^52,53^. Tobacco use showcases divergent effects between the two main subtypes of IBD. It has been associated with detrimental effects on CD, including an increased risk of disease development, progression, and suboptimal response to medical and surgical treatments. In contrast, it exhibits protective effects in UC. The underlying mechanisms are intricate^24,25^. Nicotine, an active component of tobacco, is thought to inhibit cytokines like IL-1β and IL-8, and smoking may impact smooth muscle function, affect endothelial function through nitric oxide production, and influence gut barrier integrity^54^. However, trials investigating nicotine replacement therapy in UC have not yielded compelling outcomes^55^. Moreover, a multitude of studies has unveiled a heightened prevalence or incidence of conventional risk factors, including conditions like diabetes and hypertension, among individuals with IBD when compared to their healthy counterparts^17,38,50,56^. These observed patterns of risk factor elevation align with the mechanisms detailed earlier, providing a crucial foundation to bolster a comprehensive strategy for the prevention of ASCVD within the IBD patient population.

**Figure 2:**
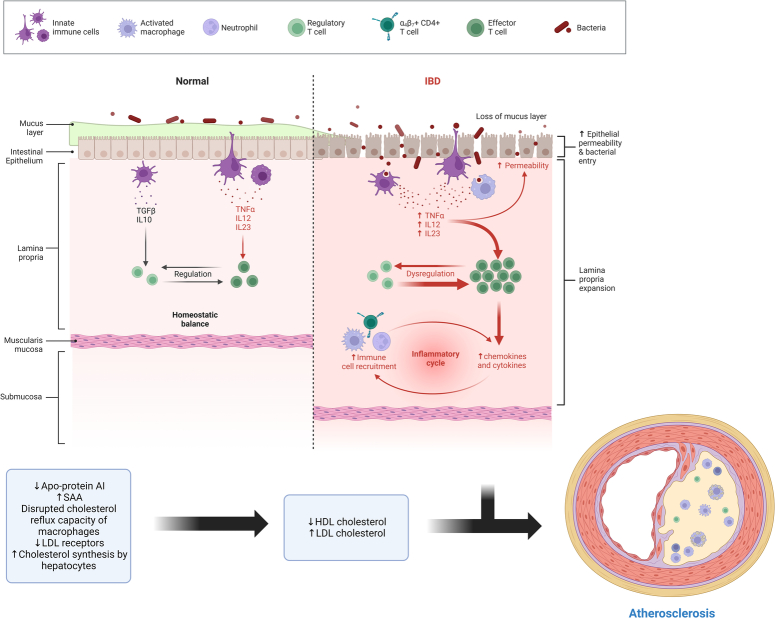
Immune cascade in inflammatory bowel disease (IBD) for atherosclerotic cardiovascular disease risk. HDL, high-density lipoprotein; LDL, low-density lipoprotein; SAA, serum amyloid protein A.

These findings not only emphasize the intricate interplay of the mechanisms discussed but also underscore the intricate relationship between IBD and ASCVD. This intricate connection highlights the necessity for multifaceted approaches that address both the underlying mechanisms driving inflammation and cardiovascular risk, as well as the shared risk factors that contribute to the development of these conditions. In essence, by comprehensively addressing these interconnected factors, there lies the potential to effectively mitigate the heightened cardiovascular risk that individuals with IBD face. This underscores the importance of personalized and targeted interventions that encompass lifestyle modifications, appropriate medical management, and close monitoring to tailor the prevention of ASCVD specifically for this unique patient population. Figure 2 shows the mechanism of the potential link between IBD and ASCVD.

### Risk assessment

At present, there exists a dearth of conclusive evidence derived from interventional studies that can definitively outline the optimal strategies for preventing ASCVD in individuals with IBD. Notably, primary prevention guidelines for ASCVD developed by prominent institutions like the American College of Cardiology (ACC) and the American Heart Association (AHA), as well as the European Society of Cardiology (ESC), acknowledge the significance of chronic inflammatory conditions as independent factors contributing to ASCVD. These guidelines advise the consideration of these conditions as risk enhancers or modifiers, thereby strengthening the case for implementing chronic statin therapy among patients who are at the cusp of borderline or intermediate risk for ASCVD^57,58^.

Further recommendations regarding risk assessment and management in individuals with chronic inflammatory conditions, including IBD, are condensed in Table 1. However, several factors that pertain specifically to the assessment of cardiovascular risk in IBD patients warrant careful consideration. Typically, patients with IBD are young adults, often falling below the threshold for undergoing a 10-year ASCVD risk assessment. Consequently, these individuals may not be evaluated or categorized accurately based on their chronological age. Furthermore, certain mechanisms by which IBD heightens ASCVD risk might not be adequately accounted for by conventional clinical risk scores. This could lead to an underestimation of their true risk profile. For instance, widely used tools such as the pooled cohort equations or the SCORE charts may not have been specifically validated to predict risk accurately in adults with IBD.

**Table 1:** Risk assessment and

Diagnosis of IBD	IBS and IBS should be differentiated by experienced physicians before commencement of treatment
Disease activity	Relevant biomarkers of disease activity should be consulted every 3 months or according to active disease status
Cardiovascular risk assessment	First line risk assessment tool should be coronary CT or MRI
Start of treatment	Prediction of endoscopic and/or histological disease activity should be taken into account
Remission	Prediction of recurrence should be explained to the patients

CT, computed tomography; IBD, inflammatory bowel disease; IBS, irritable bowel syndrome.

Given these intricacies, it becomes evident that specialized guidance and validated risk stratification tools are imperative when dealing with this distinctively youthful patient demographic. Unlike older populations, guidelines fail to address the nuances of risk heterogeneity amongst patients with inflammatory conditions like IBD. Similarly, there’s a dearth of advice regarding the utilization of supplementary risk assessment tools that could personalize risk management strategies for this particular group. A notable example is the discussion within the 2019 ACC/AHA document, which acknowledges the uncertainty surrounding the prognostic implications of a coronary artery calcium score of zero—a potent negative risk indicator in the general population—within the context of patients with inflammatory conditions^58^.

### Coronary calcium score in IBD

The use of coronary calcium scoring as a method for assessing the risk of CAD in individuals with IBD presents both opportunities and challenges. Coronary calcium scoring is a non-invasive imaging technique that quantifies the amount of calcium deposits in the coronary arteries, serving as an indicator of atherosclerosis and potential cardiovascular risk^59^. This approach is commonly employed to identify an individual’s risk of developing CAD before symptoms become apparent.

For individuals with IBD, the application of coronary calcium scoring offers several promising opportunities. Firstly, it enables the early detection of subclinical atherosclerosis, allowing medical professionals to identify the presence of coronary artery calcification even in the absence of visible CAD symptoms^60^. This is particularly pertinent for individuals with IBD, who may face an elevated cardiovascular risk due to chronic inflammation characteristic of the condition. Moreover, coronary calcium scoring provides a platform for personalized risk assessment, which is crucial considering the unique risk factors and inflammatory profiles associated with IBD^61^.

Given the diverse nature of these factors, tailoring risk assessments on an individual basis becomes essential to formulate suitable preventive strategies. By combining inflammation markers and calcium scores, a more comprehensive risk evaluation can be achieved, encompassing both structural and inflammatory aspects, thus painting a clearer picture of an individual’s CAD risk. However, certain challenges need to be addressed when applying coronary calcium scoring to individuals with IBD. One primary challenge is the absence of specific guidelines for using this technique in the context of IBD. While coronary calcium scoring has a solid foundation for CAD risk assessment in the general population, adapting it for individuals with IBD might require a more nuanced approach due to the distinct characteristics of this population^62^. Furthermore, the validity of coronary calcium scoring in predicting CAD risk in individuals with IBD has yet to be fully verified. Validation studies are essential to establish the accuracy of calcium scores in predicting cardiovascular outcomes within this specific population. Moreover, the impact of chronic inflammation associated with IBD on calcium deposition and subsequent risk stratification remains a topic of investigation^63^.

This method’s underlying principle lies in its ability to furnish critical insights into the existence and extent of atherosclerosis within the coronary arteries, thereby aiding in the evaluation of an individual’s susceptibility to CAD.

The procedure operates on the following foundational principles: (1) Detection of calcified plaque: At its core, calcified plaque stands as a characteristic feature of atherosclerosis—a condition characterized by the accumulation of fatty deposits within the arterial walls. These deposits can potentially lead to the constriction and blockage of blood vessels. The coronary calcium scan captures detailed images of the heart and its associated coronary arteries, employing advanced CT technology. (2) Quantification of calcium: The images obtained from the scan are subjected to intricate processing and analysis to discern regions with calcified plaque within the coronary arteries. The severity of calcification is quantified utilizing a scoring system, with the Agatston score being a widely used example. This score takes into account factors such as the density and scope of the calcified plaques, attributing higher scores to instances of more pronounced calcification. (3) Calculation of coronary calcium score: The Agatston score is computed by assigning numerical values based on the density of the calcified plaque, its area of occurrence, and the number of coronary artery segments that are affected. These assigned scores are then aggregated across all relevant coronary artery segments, ultimately yielding the final coronary calcium score. The score’s magnitude is directly proportional to the quantity of calcified plaque present in the arteries. (4) Risk stratification: The computed coronary calcium score serves as the foundation for categorizing individuals into distinct risk groups, depending on the likelihood of encountering cardiovascular events. Those with higher scores generally face an elevated risk of experiencing CAD and related events, such as heart attacks. (5) Clinical utility: The coronary calcium score functions as an auxiliary tool within the context of cardiovascular risk assessment. It imparts insights that extend beyond traditional risk factors—such as age, blood pressure, cholesterol levels, and smoking history. By integrating the coronary calcium score with these conventional risk indicators, the accuracy of risk prediction is significantly enhanced. This is especially pertinent for individuals whose risk might be classified as intermediate based solely on traditional factors^64^.

The coronary calcium score plays a significant role in assessing the risk and predicting the likelihood of developing ASCVD, which includes conditions like CAD, and other cardiovascular events. This scoring system is a valuable tool that helps clinicians make informed decisions about preventive measures and management strategies for individuals at risk of ASCVD. The role of the coronary calcium score in relation to ASCVD is multifaceted and can be summarized as follows:

Early detection of atherosclerosis: Atherosclerosis is a progressive condition characterized by the accumulation of plaque within the arteries, including the coronary arteries that supply the heart. The coronary calcium score provides a means of identifying the presence of calcified plaque in these arteries even before symptoms of ASCVD become evident. This early detection allows for timely interventions and preventive measures. The coronary calcium score quantifies the amount of calcified plaque present in the coronary arteries. A higher score indicates a greater burden of calcified plaque, which is associated with an increased risk of ASCVD. This quantitative assessment provides a more accurate and objective measure of an individual’s risk compared to traditional risk factors alone. Incorporating the coronary calcium score into cardiovascular risk assessment enhances risk stratification. Individuals with higher scores are categorized into higher-risk groups, indicating a greater likelihood of experiencing cardiovascular events. This allows healthcare professionals to allocate appropriate resources and interventions to those at the highest risk. The coronary calcium score contributes to the refinement of treatment strategies. Individuals with elevated scores may benefit from more aggressive interventions, lifestyle modifications, and pharmacological therapies to reduce their risk of ASCVD. Conversely, those with lower scores may require less intensive interventions. By considering the coronary calcium score along with traditional risk factors, healthcare providers can offer personalized risk assessments. This personalized approach takes into account an individual’s unique risk profile, which is particularly relevant for individuals who fall into the intermediate-risk category based solely on traditional risk factors. The coronary calcium score can be used for longitudinal monitoring of atherosclerosis progression. Periodic rescoring can help track changes in plaque burden and assess the effectiveness of interventions over time. The coronary calcium score empowers individuals to make informed decisions about their cardiovascular health. Individuals with elevated scores can better understand their risk and collaborate with healthcare professionals to develop tailored prevention and management strategies.

The link between IBD and coronary calcium score lies in the complex interplay between chronic inflammation, systemic effects, and the risk of cardiovascular disease, including atherosclerosis^15^. While the direct relationship between IBD and coronary calcium score is still being explored, several key factors contribute to their connection. IBD, characterized by chronic inflammation of the gastrointestinal tract, is associated with elevated levels of inflammatory markers throughout the body. Chronic inflammation is a known contributor to atherosclerosis—the buildup of plaque in the arteries. Inflammation can damage the arterial walls, leading to the deposition of lipids and the formation of calcified plaques, which are detected by the coronary calcium score^21^.

IBD is not confined to the digestive system; it has systemic effects. Inflammation and immune responses in IBD can impact various body systems, including the cardiovascular system^37^. Chronic inflammation in IBD might contribute to endothelial dysfunction, dyslipidemia, and other processes that promote atherosclerosis. Shared Risk Factors: IBD and cardiovascular disease share common risk factors such as smoking, sedentary lifestyle, obesity, and certain genetic predispositions. These factors can influence both the development of IBD and the progression of atherosclerosis, influencing the coronary calcium score. IBD is associated with alterations in the gut microbiome, which can affect systemic inflammation and potentially influence cardiovascular health. Emerging research suggests that the gut microbiome plays a role in cardiovascular risk through various mechanisms, including inflammation. There’s emerging interest in investigating the potential mechanisms that might link IBD to increased coronary calcium scores. These could include chronic inflammation-mediated endothelial dysfunction, changes in lipid profiles, and the systemic effects of inflammatory cytokines that could contribute to atherosclerosis.

## Other modalities for CV risk assessment

### Magnetic resonance imaging

The evolution of MRI in cardiovascular risk assessment has been a remarkable journey with several distinct stages. In the early days, during the 1980s, cardiac MRI was in its infancy and primarily utilized for research purposes^65,66^. The imaging techniques were slow and produced low-resolution images. These advancements improved the accuracy of diagnosing myocardial infarction and detecting early signs of cardiac diseases.

During the 2000s, MRI began to establish itself as a valuable tool for assessing cardiac function. This included the measurement of ejection fraction, ventricular volumes, and the detection of wall motion abnormalities^65^. Additionally, perfusion imaging, which involves tracking blood flow in the heart, was developed to assess myocardial ischaemia and determine the severity of coronary artery disease^66^. Cardiac MRI found its place in clinical practice, particularly for preoperative evaluations of congenital heart diseases, cardiomyopathies, and valvular diseases^65^. The 2010s marked another phase in the evolution of cardiac MRI. Advanced techniques, such as 3D MRI, strain imaging, and late gadolinium enhancement (LGE) imaging, became more widespread, allowing for better tissue characterization^67–69^. Quantitative MRI methods, like T1 and T2 mapping, offered insights into myocardial fibrosis and oedema, aiding in the early detection of cardiac diseases^70^.

In the present day, the use of MRI in cardiovascular risk assessment has advanced to facilitate personalized medicine and risk prediction. Advanced MRI techniques have allowed for the development of personalized treatment approaches, tailored to an individual’s unique cardiac characteristics. Moreover, there is an increasing focus on integrating machine learning and AI algorithms to predict cardiovascular risk based on a combination of MRI data and clinical information. MRI is now being used to evaluate the efficacy of treatment strategies and monitor disease progression over time, aiding in risk prediction and management.

Cardiovascular risk assessment with echocardiography is a valuable tool for healthcare professionals^71^. Echocardiography, which uses ultrasound to create real-time images of the heart, plays a crucial role in evaluating heart health and assessing the risk of cardiovascular diseases^72^. It provides insights into cardiac anatomy, helping identify structural abnormalities and assess cardiac function, such as ejection fraction, ventricular volumes, and wall motion abnormalities, which are indicative of heart health^73^. Additionally, echocardiography can indirectly assess the risk of atherosclerosis by examining the carotid arteries in the neck. Detecting atherosclerotic plaques can be vital in reducing the risk of stroke and CAD^71^.

Echocardiography’s ability to evaluate blood flow patterns and velocities aids in assessing conditions like hypertension and valvular diseases, which are significant risk factors for cardiovascular diseases^74^.

### Applanation tonometry

Applanation tonometry technique involves measuring arterial pressure and arterial stiffness to assess the risk of cardiovascular diseases^75^. Arterial pressure is measured directly, typically at the radial artery, providing a precise assessment of central aortic pressure, which is a better indicator of cardiovascular risk than traditional brachial blood pressure measurements. Elevated central aortic pressure is associated with a higher risk of heart disease and stroke^76^. Arterial applanation tonometry also enables the evaluation of arterial stiffness, often measured through the pulse wave velocity. Increased arterial stiffness is a marker of vascular aging and a significant risk factor for cardiovascular diseases, including conditions like atherosclerosis, hypertension, and diabetes^77^. The technique can also determine the augmentation index, reflecting the degree of wave reflection in the arterial system^78^. A higher AI is associated with increased cardiovascular risk and is often seen in individuals with stiff arteries, contributing to elevated central aortic pressure and a greater likelihood of cardiovascular problems^78^.

### Clinical implications

The clinical implications of the relationship between IBD and coronary calcium score highlight the importance of comprehensive cardiovascular risk assessment and management for individuals with IBD. While the direct impact of IBD on coronary calcium scores is still being elucidated, several key clinical implications arise from understanding this association: (1) Enhanced cardiovascular risk assessment: Recognizing the potential link between IBD and increased cardiovascular risk, healthcare providers should consider including cardiovascular risk assessments, such as coronary calcium scoring, as part of routine care for individuals with IBD. This allows for a more accurate evaluation of a patient’s cardiovascular health beyond traditional risk factors. (2) Tailored preventive strategies: Individuals with IBD who demonstrate elevated coronary calcium scores may be at a higher risk of atherosclerosis and cardiovascular events. This information can guide healthcare professionals in tailoring preventive strategies that are more aggressive and targeted. Lifestyle modifications, such as adopting heart-healthy diets and increasing physical activity, along with pharmacological interventions, can be recommended based on individual risk profiles. (3) Informed shared decision-making: The integration of coronary calcium scores into discussions with patients fosters informed shared decision-making. Individuals with IBD can better understand their cardiovascular risk and collaborate with healthcare providers to develop personalized management plans that align with their specific needs and health goals. (4) Monitoring disease progression: Incorporating coronary calcium scoring in the care of individuals with IBD allows for ongoing monitoring of disease progression. Regular rescoring can track changes in calcified plaque burden over time, enabling timely adjustments to management strategies if needed. (5) Focus on lifestyle modifications: Recognizing the potential impact of chronic inflammation in IBD on cardiovascular health, healthcare providers can emphasize lifestyle modifications that target inflammation reduction. This holistic approach may involve dietary changes, stress management, and other measures that could positively influence both IBD and cardiovascular risk. (6) Collaborative care: The association between IBD and cardiovascular risk underscores the need for multidisciplinary care. Collaboration between gastroenterologists, cardiologists, and other relevant specialists can ensure a holistic approach to managing both IBD-related inflammation and cardiovascular risk factors. (7) Early intervention: Utilizing coronary calcium scoring can help identify individuals at higher risk of developing cardiovascular disease early on. Early intervention with appropriate preventive measures can potentially slow down or even halt the progression of atherosclerosis, reducing the risk of cardiovascular events. (8) Research opportunities: The link between IBD and coronary calcium scores presents opportunities for further research. Investigating the mechanisms underlying this association could pave the way for innovative treatments and interventions that target both IBD-related inflammation and cardiovascular risk.

### Future directions

Several significant gaps in evidence still exist within the context of the relationship between IBD and ASCVD. Addressing these gaps is crucial for gaining a more comprehensive understanding of the mechanisms underlying ASCVD development in individuals with IBD. This need is particularly evident in exploring intermediate mechanisms and early atherosclerotic changes within the coronary arteries. Such research endeavours are poised to provide valuable insights into the variability of ASCVD risk among individuals with IBD. By identifying those at the highest risk and recognizing resilient phenotypes with minimal ASCVD susceptibility, tailored preventive strategies can be optimized. Moreover, delving deeper into these mechanisms will facilitate the creation of specialized risk assessment tools. Understanding the intricate interplay between IBD-related inflammation and ASCVD will empower healthcare providers to predict cardiovascular risk more accurately in this patient population. This extends to considering the potential utility of cardiovascular imaging tools, such as computed tomographic angiography, in quantifying coronary plaque burden and identifying plaque characteristics linked to elevated ASCVD risk. Particularly in individuals with IBD who are prone to increased plaque burden and high-risk plaque features, the potential of these imaging techniques to enhance the identification of candidates for intensive ASCVD preventive interventions beyond traditional risk factors and the coronary calcium score warrants thorough evaluation. The avenue of randomized trials holds great promise in shedding light on various aspects of the IBD-ASCVD relationship. These trials can provide deeper insights into the potential role of IBD-modifying therapies in mitigating ASCVD risk. By studying the impact of interventions that target key pathophysiological mechanisms—such as lipid dysfunction and prothrombotic state—researchers can unlock essential information regarding their efficacy in reducing cardiovascular risk, particularly among individuals at the highest risk of ASCVD. Additionally, assessing the correlation between these interventions and coronary calcium scores can further elucidate their potential to influence atherosclerotic burden and plaque characteristics.

### Limitations

The review of the relationship between (IBD and CVD confronts several limitations inherent in the current body of evidence. Primarily based on observational and heterogeneous studies, the establishment of causality between IBD and heightened CVD risk remains challenging. Insufficient mechanistic comprehension impedes the development of targeted interventions. The dearth of interventional studies tailored for individuals with IBD hampers the formulation of evidence-based guidelines for managing CVD risk in this population. The scarcity of longitudinal studies tracking CVD outcomes over time in IBD patients and the necessity for validated risk assessment tools specific to this demographic underscore the limitations in risk prediction and understanding long-term cardiovascular trajectories.

## Conclusion

In conclusion, the intricate relationship between IBD and ASCVD reveals a tapestry of connections that have far-reaching implications for healthcare. The synergy between chronic inflammation in IBD and the heightened risk of ASCVD underscores the need for precision in risk assessment. The coronary calcium score, through its ability to quantify calcified plaque within coronary arteries, presents an opportunity to augment traditional risk factors. This integration provides a more comprehensive understanding of cardiovascular risk for individuals with IBD, enabling healthcare providers to tailor interventions based on a nuanced assessment of their ASCVD susceptibility. The potential of the coronary calcium score extends beyond risk assessment—it holds the promise of informing targeted interventions. Through randomized trials, we can unveil the impact of IBD-modifying therapies on ASCVD risk reduction, paving the way for more nuanced treatment approaches. This becomes particularly pertinent for individuals with IBD who are at the highest risk, where understanding the value of interventions targeting specific pathophysiological mechanisms, in conjunction with the coronary calcium score, can revolutionize their cardiovascular care.

## Ethical approval

Ethics approval was not required for this Review Article.

## Consent

Informed consent was not required for this Review Article.

## Source of funding

The authors received no specific funding for this manuscript.

## Author contribution

Concept: J.M., H.A., N.I., V.K., U.N.K. Data collection: C.P.K., F.P., H.A. Design: S.K., M.T.H., D.K., D.R., F.P. Data analysis: S.K., A.M. Writing: C.P.K., F.P., H.A., S.K., M.T.H., D.K., D.R., F.P., S.K., J.M. Supervision: A.M., J.M.

## Conflicts of interest disclosure

The authors declare no conflict of interest.

## Research registration unique identifying number (UIN)

None.

## Guarantor

Amin mehmoodi.

## Data availability statement

None.

## Provenance and peer review

Not invited.
